# Pathways between Risk/Protective Factors and Maternal Postnatal Depressive Symptoms: The ELFE Cohort

**DOI:** 10.3390/jcm12093204

**Published:** 2023-04-29

**Authors:** Mélanie Bales, Elodie Pambrun, Charlotte Maguet, Judith van der Waerden, Nine Glangeaud-Freudenthal, Marie-Aline Charles, Corinne Bois, Maria Melchior, Jeannette Milgrom, Bruno Falissard, Hélène Verdoux, Anne-Laure Sutter-Dallay

**Affiliations:** 1 Inserm, Bordeaux Population Health Research Center, U1219, Bordeaux University, 33000 Bordeaux, France; 2 University Department of Child and Adolescent Psychiatry, Charles Perrens Hospital, 33076 Bordeaux, France; 3 Institut Pierre Louis d’Epidémiologie et de Santé Publique, Équipe de Recherche en Épidémiologie Sociale, INSERM U1136, Sorbonne Université, 75012 Paris, France; 4 INSERM Obstetrical, Perinatal and Pediatric Epidemiology Research Team, Center for Epidemiology and Biostatistics (U1153), Paris Descartes University, 75014 Paris, France; 5 INED, INSERM EFS, Joint Unit ELFE, 75004 Paris, France; 6 Parent-Infant Research Institute, Melbourne School of Psychological Sciences, University of Melbourne, Melbourne 3010, Australia; 7 CESP/INSERM 1018 (Centre de Recherche en Épidémiologie et Santé des Populations), Maison de Solenn, 75619 Paris, France

**Keywords:** maternal mood disorders, pathways, risk and protective factors

## Abstract

Objective: The risk factors for postnatal depressive symptoms (PNDS) are numerous, but little is known about the protective factors or the interactions between different exposures. The present study explored the pathways between maternal, infant and parenthood vulnerabilities or risk/protective factors and PNDS at 2 months postpartum (PP) in a large sample of women from the general population. Methods: We used data from the French ELFE cohort, a nationally representative cohort of children followed-up from birth. The available information about vulnerabilities or risk/protective factors for PNDS was collected during the maternity ward stay (mother or medical records) and at 2 months PP (mother by phone). PNDS were evaluated with the Edinburgh Postnatal Depression Scale (EPDS) at 2 months. A measurement model was built based on the psychosocial model for PNDS of Milgrom and colleagues using exploratory factor analysis. The Structural Equation Model was used to investigate the pathways between vulnerability, risk/protective factors and PNDS at 2 months PP. Results: In the study sample (*n* = 11,583), a lack of a partner’s perceived antenatal emotional support, consultation with a mental health specialist before pregnancy, family financial difficulties, prenatal psychological distress and a difficult pregnancy experience were directly associated with the severity of maternal PNDS at 2 months PP, as well as lack of perceived postnatal support. Family financial difficulties and consultation with a mental health specialist before pregnancy were also indirectly associated with the intensity of PNDS through a lack of perceived antenatal emotional support, a difficult pregnancy experience, prenatal psychological distress and a lack of perceived postnatal support. Regarding infant and parenthood characteristics, infant self-regulation difficulties, maternal difficulty in understanding infant crying and infant hospitalisation were directly associated with PNDS severity at 2 months PP, while maternal difficulty in understanding an infant’s cries was also indirectly associated with infant self-regulation difficulties. Conclusions: Perinatal professional support should begin antenatally and target the couple’s prenatal functioning, with particular attention to women presenting a history of psychiatric disorders, especially those of low socioeconomic status. After delivery, addressing infant and parenthood characteristics is also recommended.

## 1. Introduction

Maternal mood disorders are among the most frequent, burdensome and costly health problems during the perinatal period [1]. A large body of research has explored the risk factors of maternal postnatal depressive symptoms (PNDS). A history of depression is currently the main identified risk factor for PNDS [2]. Stressful life events and obstetrical complications also contribute to an increased risk of presenting with clinically significant PNDS [3]. On the other hand, variations in social and partner support, socioeconomic status (SES), maternal personality traits and infant temperament may function as either risk or protective factors [4,5,6,7,8,9]. Finally, all these factors may influence the occurrence of PNDS throughout the perinatal period. However, few studies have explored the interactions between maternal and infant characteristics and environmental factors in the postpartum period [10] and, more specifically, through biopsychosocial models of PNDS, as conceptualised by Engel (1977) [11]. The rare existing studies in this area were mostly focused either on one or a limited number of specific risk factors [12,13,14,15,16,17,18] or were restricted to the postpartum period [12,18,19]. Furthermore, to our knowledge, none of these studies explored the impact of potentially protective factors, as has been proposed by one of the pioneering biopsychosocial models for the understanding of postpartum depression [20]. Finally, past studies were primarily based on small samples with limited representativeness [12,13,17,18,21,22].

The present study aimed to explore the pathways between a wide range of risk and protective maternal, infant and environmental factors present both before and after birth and the severity of PNDS at 2 months postpartum in a large sample of women from the general population.

## 2. Materials and Methods

### 2.1. Data Source

The study data originated from the French nationally representative ELFE (Etude Longitudinale Française depuis l’Enfance) cohort, designed to follow up on infant and child development in their environment. The protocol, design and recruitment procedures of the ELFE survey have been previously described [23]. Briefly, women giving birth in 349 randomly selected maternity hospitals among the 544 French metropolitan public and private maternity hospitals were recruited for four to eight days in each of the four quarters of 2011. Mothers were eligible for data collection if they fulfilled the following criteria: (1) live birth; (2) term > 33 weeks; (3) single or twin pregnancy; (4) mothers aged 18 years and over; (5) understanding the main implications of the study in one of the following languages: French, English, Arabic, Turkish; (6) living and planning to stay in France for at least 3 years; and (7) giving their written informed consent for data collection. Overall, 18,329 mothers were included.

The baseline assessment took place during the maternity hospital stay. Midwives collected information from the mother’s medical records and during a face-to-face interview with the mothers using a paper questionnaire. The second assessment took place between 6 and 8 weeks postpartum with 90% of the included families participating. Trained investigators collected maternal and infant information from the mother with a questionnaire during a telephone interview. Only mothers living with a partner who was also the father of the child, and with no missing data for the variables of interest, were included in the present study.

The ELFE cohort received ethical approval from bodies overseeing data collection procedures in France (the Committee for the Protection of Persons, the National Consultative Committee for the Processing of Information in the Health Sector and the French National Data Protection Authority—CNIL).

### 2.2. Theoretical Model

**Figure 1:**
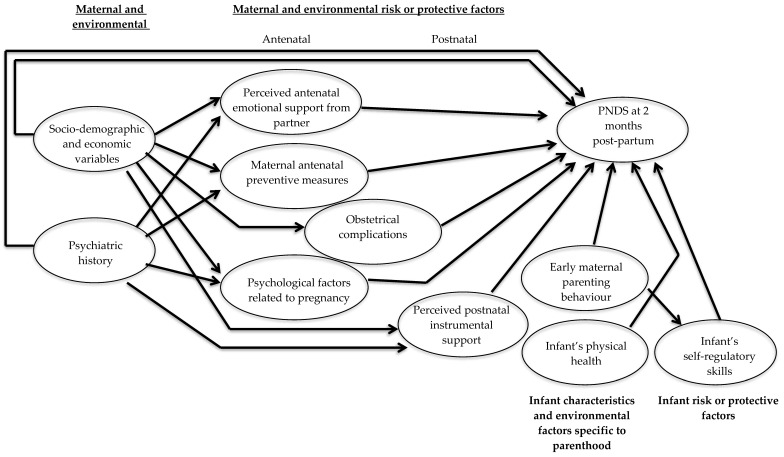
Theoretical model of intensity of PNDS.

The present study is based on a theoretical model that was developed based on pre-existing studies and models (Figure 1), particularly Milgrom’s biopsychosocial model for postpartum depression [20], which emphasises the importance of protective factors. The present model was adapted to the data available in the ELFE cohort, which was not designed for the specific purpose of exploring predictors of maternal PNDS. Nevertheless, it offers the opportunity to explore, in a general population setting, the direct and indirect links between a wide range of maternal and environmental pre and postnatal data, as well as infant and parenthood characteristics and the severity of the mother’s postnatal depressive symptoms at 2 months postpartum.

### 2.3. Assessment of Postnatal Depressive Symptoms (PNDS)

The Edinburgh Postnatal Depression Scale (EPDS) [24] was used between 6 and 8 weeks postpartum to evaluate the intensity of PNDS. The EPDS is a widely used 10-item self-reported questionnaire aimed at screening for PNDS, and it has been translated and validated for French-speaking populations [25,26]. The scores (ranging from 0 to 30) can be used as a categorical variable, with a variable cut-off depending on the purpose (>11 for screening, >12 for research) [25,26] or as a continuous score [27]. In this study, we used the continuous score because sub-syndromal cases of postnatal depression can also have a significant impact on the mother’s well-being and mother-infant interactions [28].

### 2.4. Maternal and Environmental Vulnerability Factors

For the present study, we selected variables available in the ELFE cohort and considered by the literature as vulnerability factors for the development of postnatal depressive symptoms. We selected 6 socio-demographic and economic variables collected from the baseline face-to-face interview during the hospital maternity stay or from the postpartum telephone interview (6–8 weeks postpartum): (1) mother’s age; (2) mother’s nationality; (3) mother’s educational level; (4) mother’s employment status during pregnancy; (5) number of children; and (6) family financial difficulties. The details about these variables are presented in Table 1.

We also selected 2 variables reflecting maternal psychiatric history collected from the baseline face-to-face interview during the hospital maternity stay or from the postpartum telephone interview (6–8 weeks postpartum): (1) consultation with a mental health specialist (psychiatrist, psychologist, psychotherapist or another doctor) before pregnancy and (2) self-reported depressive symptoms or an episode during a previous pregnancy.

### 2.5. Maternal and Environmental Risk or Protective Factors

We selected the variables available in the ELFE study and considered in the literature as possibly influencing maternal postnatal mood, whether positively or negatively. They were divided into 5 groups based on the reports in the literature [4,5,6,7,8,9].: (1) perceived antenatal emotional support from the partner; (2) perceived postnatal instrumental support; (3) maternal antenatal preventive measures; (4) obstetrical complications (OCs); and (5) psychological factors related to the pregnancy experience. Information regarding maternal antenatal preventive measures (early prenatal interview, antenatal classes) was collected from the mother’s face-to-face interview during the maternity hospital stay. Information about perceived antenatal emotional support (antenatal emotional support from the partner, quarrels with or without insults within the couple) and perceived postnatal instrumental support (for baby’s care and household chores) were collected from the mothers during the postpartum phone interview. The occurrence of OCs was listed based on the information collected from the maternal and child medical records, categorised as complications during pregnancy/at birth or neonatal complications and ranked according to the McNeil-Sjöström scale, a 6-point severity scale ranging from 1 (not harmful or relevant) to 6 (very great harm or deviation in offspring) [29]. The highest severity level complications were selected for each mother for each of these 2 categories. We considered that a mother presented an OC when she had at least one OC from level 3 or higher. The absence of complications was also considered a category. Finally, the psychological factors included 2 items collected from mothers during a face-to-face interview during the hospital maternity stay (reaction about the current pregnancy, declared prenatal psychological distress) and 2 items collected from mothers during the postpartum telephone interview (desire for pregnancy and experience of pregnancy). The details about these variables are presented in Table 1.

### 2.6. Infant Characteristics and Environmental Factors Specific to Parenthood

The infant and environmental factors specific to parenthood that were likely to influence maternal PNDS and were available in the ELFE were divided into 2 groups of variables: (1) early maternal parenting behaviour and (2) the infant’s physical health. Information about maternal parenting was recorded from the mother during the postpartum telephone interview through 5 items: sing-song, talking to the child, maternal ability to understand the infant’s cries, respect for the infant’s feeding rhythm and the mother’s reaction to her baby eating too little (if breastfed) or not finishing his/her bottle (without illness context). Information about the infant’s physical health was recorded from the mother during the postpartum telephone interview through 2 items: the infant’s condition evaluated by the mother and the infant’s hospitalisation since returning from the maternity hospital stay. The details about these variables are presented in Table 1.

### 2.7. Infant Risk or Protective Factors

Infant risk/protective factors were defined by a single group of variables, namely the infant’s self-regulation skills: (1) self-appeasement; (2) frequency of crying; and (3) nocturnal awakenings. They were collected from mothers during the postpartum telephone interview. The details are presented in Table 1.

### 2.8. Statistical Analyses

A two-step approach was taken in the analyses. In the first step, a measurement model, which tests how well the chosen indicator variables measure the latent constructs, was established. The items of each group of maternal and infant variables were subjected to exploratory factor analysis (EFA). The resulting EFA solutions were reviewed, taking care to adhere to the recommended criteria, including an assessment of eigenvalues and ensuring the factor loadings were salient at greater than 0.30. If no latent factor is found, researchers may decide to keep the variables as measured variables based on the literature on the topic. In the second step, we implemented a structural equation modelling (SEM) analysis using weighted least squares (WLS) estimation. This estimation method makes it possible to overcome the assumption of multinormality of the variables and requires a sample of substantial size [30]. SEM allows the testing of simultaneous direct and indirect pathways in analyses. This method provides a reliable alternative to classical methods to investigate relationships inside a framework of measured variables and latent factors. We first tested the direct effects of each latent or measured variable on the outcome variable intensity of maternal PNDS at 2 months postpartum. The covariances among the maternal and environmental factors were modelled as well as among the infant characteristics and environmental factors specific to parenthood and also between these two groups of variables. The variables that were not associated were removed, and the model was tested again with the remaining variables. Then, we analysed the potential mediating effects of maternal risk/protective factors for PNDS and infant risk/protective factors for PNDS at 2 months postpartum with the Baron and Kenny approach and Sobel’s Test [31]. This test is one of the most used and can be used in particular in large samples. The comparative fit index (CFI) was used as a measure of fit of the models. Values greater than approximately 0.90 indicate a reasonably good fit of the SEM model. The root mean square error of approximation (RMSEA) was used to assess the error of approximation. An RMSEA ≤ 0.05 indicates a close approximate fit, and values between 0.05 and 0.08 suggest a reasonable error of approximation. The analyses were performed using SAS [32] (SAS Institute Inc., 2011) and AMOS [33] (Arbuckle, 1999).

## 3. Results

### 3.1. Characteristics of the Sample

**Figure 2:**
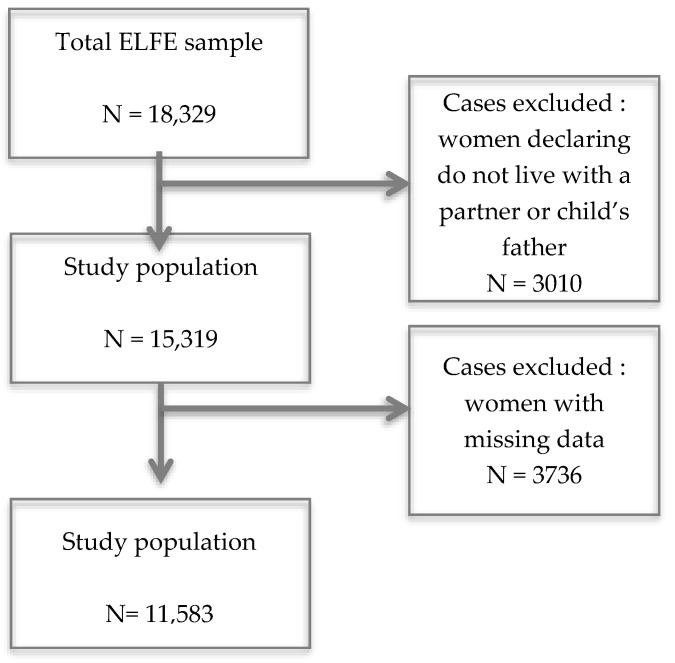
Flow chart showing sample derivation.

The sample studied in the present work was restricted to the 11,583 mothers living with a partner and with no missing data for the variables of interest (Figure 2). The maternal and environmental vulnerability characteristics of the sample are presented in Table 2 (More details of the characteristics of the sample (Table S1) and the characteristics of women with missing data (Table S2) are available in the Supplementary Material).

### 3.2. EFA

In the present study, the optimal EFA solution showed four latent factors: a lack of perceived antenatal emotional support from the partner (antenatal emotional support from the spouse, quarrels with or without insults within the couple); a lack of perceived postnatal instrumental support in the baby’s care (changing diapers, feeding, washing, putting him/her to sleep, getting up in the night because of cries); a lack of perceived postnatal instrumental support in household chores (washing dishes, doing laundry, doing the housework, repairs); and infant self-regulation difficulties (self-appeasement, frequency of crying). For the other groups of variables, no latent factors were found. The literature on the topic [4,6,7] allowed us to keep the following variables as measured variables: mother’s age and family financial difficulties (from demographic and socioeconomic variables); consultation with a mental health specialist and depression during a previous pregnancy (from psychiatric history variables); complications during pregnancy (from obstetrical factors); difficult experience of pregnancy (from psychological factors); prenatal psychological distress (from psychological factors); maternal difficulty in understanding an infant’s cries (from early maternal parenting behaviour); and infant hospitalisation since returning from the maternity hospital stay (from infant’s physical health). The item did not attend prenatal care was conserved as a measured variable. After the EFA, the model included 4 latent factors and 10 measured variables. The details of the EFA are available in the Supplementary Material (Tables S3–S16).

### 3.3. Structural Equation Modeling

Fully standardised estimates indicate that three latent factors (a lack of perceived antenatal emotional support (β = 0.31, *p* < 0.001), a lack of perceived postnatal instrumental support in caring for the baby (β = 0.05, *p* < 0.001) and infant self-regulation difficulties (β = 0.20, *p* < 0.001)) and six measured variables (family financial difficulties (β = 0.08, *p* < 0.001), consultation with a mental health specialist (β = 0.09, *p* < 0.001), a difficult experience of pregnancy (β = 0.03, *p* < 0.01), prenatal psychological distress (β = 0.05, *p* < 0.001), infant hospitalisation since returning from the maternity hospital stay (β = 0.03, *p* < 0.001) and maternal difficulty in understanding an infant’s cries (β = −0.06, *p* < 0.05)) were directly associated with the intensity of maternal PNDS at 2 months postpartum. The latent factor a lack of postnatal instrumental support in household chores (β = 0.01, *p* = 0.49), as well as three measured variables: mother’s age (β = −0.01, *p* = 0.49), depression during a previous pregnancy (β = 0.02, *p* = 0.06) and complications during pregnancy or at birth (β = −0.01, *p* = 0.11), were not significantly associated with the outcome variable and were removed from the model.

**Figure 3:**
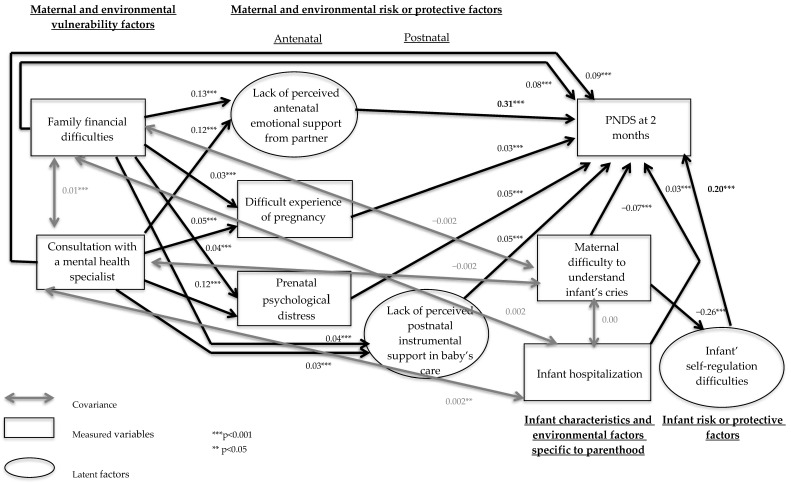
Final model of intensity of depressive symptomatology.

Two non-significant direct pathways were also removed from the model. Consultation with a mental health specialist was not significantly associated with a lack of perceived postnatal instrumental support in caring for the baby and infant hospitalisation since returning from the maternity hospital stay was not significantly associated with infant self-regulation difficulties. Figure 3 shows the final model with only the significant pathways between the remaining variables.

The results of Sobel’s Test indicate that the relationship between family financial difficulties and PNDS at 2 months postpartum was significantly mediated by a lack of perceived antenatal emotional support (Z = 7.14, *p* < 0.001), a lack of perceived postnatal instrumental support in caring for the baby (Z = 3.20, *p* < 0.001), a difficult experience of pregnancy (Z = 4.89, *p* < 0.001) and prenatal psychological distress (Z = 4.20, *p* < 0.001). The relationship between consultation with a mental health specialist and PNDS at 2 months postpartum was significantly mediated by a lack of perceived antenatal emotional support (Z = 6.15, *p* < 0.001), a difficult experience of pregnancy (Z = 5.86, *p* < 0.001) and prenatal psychological distress (Z = 5.87, *p* < 0.001). The relationship between maternal difficulty in understanding an infant’s cries and PNDS at 2 months postpartum was significantly mediated by infant self-regulation difficulties (Z = −8.94, *p* < 0.05). In contrast, the relationship between infant hospitalisation since returning from the maternity hospital stay and PNDS at 2 months postpartum was not significantly mediated by infant self-regulation difficulties. Maternal antenatal preventive measures did not significantly mediate the relationship between familial financial difficulties or consultation with a mental health specialist and PNDS at 2 months postpartum, so it was removed from the model.

The final model explained 19% of the variance in the intensity of maternal PNDS at 2 months postpartum. The results of the path analyses displaying the standardised coefficients for each of these pathways are presented to further illustrate each of the above relationships (Figure 3).

## 4. Discussion

By testing a psychosocial model of PNDS in a low-risk general population sample of new mothers living with a partner, from pregnancy to 2 months postpartum, we found effects of both the maternal and infant characteristics.

In detail, regarding the maternal and environmental prenatal variables, and following a decreasing effect size, a lack of perceived antenatal emotional support from the partner, consultation with a mental health specialist before pregnancy, family financial difficulties, prenatal psychological distress and a difficult experience of pregnancy were positively and directly associated with the severity of PNDS at 2 months postpartum. Postnatally, a lack of perceived postnatal support in the baby’s care was positively and directly associated with the severity of PNDS. Family financial difficulties and consultation with a mental health specialist before pregnancy were also positively and indirectly associated with the intensity of PNDS, through a lack of perceived antenatal emotional support from the partner, prenatal psychological distress, a difficult experience of pregnancy and a lack of perceived postnatal support in the baby’s care.

Regarding infant and parenthood dimensions, our results show a positive direct association between infant self-regulation difficulties, infant hospitalisation, and the intensity of PNDS at 2 months postpartum, and a negative direct association between maternal difficulty in understanding an infant’s cries and the intensity of PNDS at 2 months postpartum. Maternal difficulty in understanding an infant’s cries was also negatively and indirectly associated with the intensity of PNDS through infant self-regulation difficulties.

The results of this study first underline the crucial role of support during the perinatal period, especially from the partner. The impact of a partner’s support is already well documented in terms of the impact on PNDS [34,35,36,37,38]. However, our results highlight, in line with some other studies, that more than postnatal current partner support, the quality of the couple’s relationship during pregnancy may have a direct protective effect on maternal postnatal emotional stability [8]. Thus, when current preventive interventions for postnatal depression mainly target a mother’s emotional health, our results suggest, in line with some other studies [39], that the quality of the couple’s relationship should also be targeted during preconceptional and pregnancy consultations. These results also highlight the importance of the father’s mental health during the perinatal period.

Having had a consultation before pregnancy for a mental health problem was positively and directly associated with the intensity of PNDS at 2 months postpartum. This result is consistent with past results showing that a history of psychiatric problems is one of the main risk factors for PNDS [4,6,7]. In addition, our results show that in women with a history of psychiatric disorders, the intensity of PNDS is mediated by pre and postnatal support, prenatal psychological distress and the experience of pregnancy. These results underline once more, first, the importance of being aware of a woman’s psychiatric history as early as possible—when a pregnancy is planned or at the early stages—to adapt the healthcare pathways and second, that for the pathways to be effective, all the associated vulnerabilities, as well as the emotional status all along the pregnancy, must be considered.

Past studies exploring the associations between familial financial difficulties and maternal perinatal emotional stability revealed weak associations [4,6,7]. By exploring family financial difficulties as part of a pathway model, our study underlines not only its direct impact on the intensity of PNDS but also its impact through associated vulnerability factors. The mediating effects of a lack of perceived antenatal and postnatal support, prenatal psychological distress and a difficult experience of pregnancy highlight the core role of the quality of perinatal support and psychological factors among socioeconomically vulnerable women [40]. Furthermore, beyond its effect on maternal mental health, a low SES is also a risk factor for maternal, foetal and infant global morbidity [41,42], which underlines the absolute necessity to pay special attention to low SES pregnant women and the need for very early and targeted interventions, especially in the most vulnerable women and their infants.

Regarding infant and parenthood-related factors, up to now, most of the research was focused on the impact of maternal depression on infant and child development. Few studies have explored the impact of the infant’s behavioural characteristics on the occurrence of PNDS in the mother. To our knowledge, only one study explored a few postnatal variables among a small sample of mothers and their babies using SEM [43], and a strong direct association between the frequency of a baby’s cries and maternal PNDS at 3 months postpartum was observed. Vik et al. (2009) found an independent association between prolonged crying in babies and the intensity of PNDS at 2 and 6 months postpartum [44]. Finally, a study conducted by Eastwood et al. (2012) showed independent associations between a baby’s behavioural characteristics (“demanding” or “difficult to comfort”) and maternal PNDS in a large general population of mothers and their infants [45]. The results of the present study are in line with these few preceding studies and underline the direct impact of a baby’s self-regulation skills on maternal postnatal emotional stability. It is likely that infant self-regulation difficulties affect the quality of the interaction between the baby and the mother, with a possible consequent loss of confidence in her parenting abilities, impacting her mood. However, these results must be interpreted cautiously because the infant self-regulation skills were evaluated through the mothers’ reports during the same sequence of the study, and a mother presenting with depressive symptoms might tend to evaluate more negatively the capacity of her baby to self-regulate.

Finally, infant hospitalisation was positively and directly associated with the intensity of PNDS. An infant’s hospitalisation and/or the presence of health problems are well-recognised stress factors for the mother, leading to an increased risk for the mother presenting with PNDS [46]. Here, even in a low-risk sample of infants and parents, our results suggest the need for health professionals to pay special attention to all parents of hospitalised babies.

Two negative findings are of interest. First, the associations between OCs and PNDS were not significant. This suggests that they would not be a key variable in understanding the emotional stability of postpartum women, or at least not in the general population. Similarly, prenatal maternal preventive measures were not a significant variable, which highlights that these measures alone are not sufficient, especially for vulnerable women.

Our study presents limitations. The first is that the studied sample excluded mothers with missing data. This may have resulted in our sample presenting women at lower risk of mental disorders, especially mood disorders (more likely to be 25–35 years, being French, being employed, with a high educational level, without financial difficulties). Another limitation is the fact that the mother herself provided the infant’s information, leading to a possible measurement bias. The reason for that is that the ELFE study was aimed at addressing many topics related to health, the environment and socialisation in a large cohort of children in the general population, making it necessary to obtain a large amount of information. Thus, the use of simple and broad questions was mandatory, as for many other large cohort studies [47,48].

The present study also has strengths by being, to our knowledge, the first to explore the pathways between a wide range of vulnerability, risk and protective factors and the intensity of PNDS at 2 months postpartum in a large birth cohort. It highlights the critical need to work with multifactorial and longitudinal models in research on maternal perinatal mental health and infant development. Indeed, they probably represent one of the best strategies for exploring the links between the many ante- and postnatal factors and, above all, are as close as possible to clinical situations and care practices.

The findings of our study imply that familial support should be better targeted prenatally on couple functioning, with a special focus on women with low economic status and/or with a history of psychiatric disorders. Postnatally, infant factors and parenting skills are potential key vulnerability factors for maternal postnatal mental health and must also be taken specifically into account in setting up prevention and follow-up programmes adapted to each situation [39,40,41,42,43,44,45,46,47,48,49]. Perinatal familial support and care could be redesigned by including all these specific goals to achieve a more significant preventive effect in the general population and the next generations. Thus, further research is needed to understand the underlying mechanisms that are associated with a higher prevalence of maternal perinatal mood disorders, in particular by including recent hypotheses on the neurobiology of depression and consideration of the neurodevelopmental nature of these disorders [50,51,52].

## Figures and Tables

**Table 1:** Detailed description of variable categories.

Maternal and Environmental Vulnerability Factors	Maternal and Environmental Risk or Protective Factors	Infant Characteristics and Environmental Factors Specific to Parenthood	Infant Risk or Protective Factors
Socio-demographic and economic variables	Perceived antenatal emotional support from partner	Early maternal parenting behaviour	Infants’ self-regulation skills
Mother’s age - 18–24 (=1) - 25–34 (=2) - ≥35 years (=3)	Antenatal emotional support from spouse - Very good support (=1) - Good support (=2) - Little support (=3) - No support (=4)	Sing songs - Every day (=1) - Sometimes (=2) - Rarely (=3)	Self-appeasement - Never cry or almost never (=1) - Quite often alone (=2) - Only if parents remain with him (=3) - Only if parents take him in arms (=4)
Nationality - French (=1) - French by naturalization (=2) - Foreigner (=3)	Quarrels with or without insults within the couple - Never (=1) - Rarely (=2) - Sometimes without insults (=3) - Sometimes with insults (=4)	Talk to the child - Every day (=1) - Sometimes (=2) - Rarely (=3)	Frequency of crying - Rarely (=1) - Often (=2) - Very often (=3)
Educational level - <9 (=3) - 9–11 (=2) - ≥12 years (=1)	Perceived postnatal instrumental support	Maternal ability to understand infant cries - Often (=3) - Sometimes (=2) - Rarely (=1)	Nocturnal awakenings - Never or almost never (=1) - Sometimes or often (=2) - All nights or almost (=3)
	For baby’s care (Changing diapers, feeding, wash, put it to sleep, taking it for a walk, getting up in the night because of cries, taking it to the doctor) - Mainly the mother (=1) - Mainly the father (=2) - Equal division of labour (=3) - Another person (=4)		
Mother’s employment status during pregnancy - Employed or student (=1) - Housewife, on parental leave or retired (=2) - Unemployed (=3)	For household chores (Washing dishes, doing shopping, preparing meals, doing laundry, doing the housework, repairs) - Mainly the mother (=1) - Mainly the father (=2) - Equal division of labour (=3) - Another person (=4)	Respect of infant’s feeding rhythm - Wakes him to feed (=3) - Requests him at regular time but only if baby is awake (=2) - Feeds him on demand (=1)	
Number of children - 1 (=1) - 2 (=2) - 3 or more (=3)	Maternal antenatal preventive measures	Mother’s reaction if baby eats little or don’t finish his bottle (without illness context) - Insists to feed him (=3) - Proposes him later (=2) - Don’t insist or it never happens (=1)	
	Attending early prenatal interview, attending antenatal classes - Attending the two (=1) - Attending one (=2) - Attending none (=3)		
Familial financial status - High and middle (=1) - Low (=2) - Very low (=3)	Obstetrical complications	Infant’s physical health	
	Complications during pregnancy - None (=1) - Level 3 (=2) - Level 4 (=3) - Levels 5 or 6 (=4)	Infant’s condition evaluated by the mother - Healthy (=1) - Rather healthy (=2) - Rather poor health or poor health (=3)	
Psychiatric History	Complications at birth, or neonatal complications - None (=1) - Level 3 (=2) - Level 4 (=3) - Levels 5 or 6 (=4)	Infant hospitalization since returning from maternity hospital stay - No (=0) - Yes (=1)	
Consultation with a mental health specialist before pregnancy (psychiatrist, psychologist, psychotherapist or another doctor) - No (=0) - Yes (=1)			
Depression during a previous pregnancy - No (=0) - Yes (=1)	Psychological factors		
	Reaction about the current pregnancy - Happy that it happened now (=1) - Ambivalent towards this pregnancy (=2) - Unwanted pregnancy (=3)		
	Experience of pregnancy - Pleasant (=1) - Pleasant with some difficulties (=2) - Difficult (=3)		
	Desire of pregnancy - Yes (=1) - With hesitations (=2) - No (=3)		
	Prenatal psychological distress - No (=0) - Yes (=1)		

( ) variable coding.

**Table 2:** Characteristics of the sample (*n* = 11,583).

Maternal and Environmental Vulnerability Factors	
Socio-Demographic and Economic Variables	*n* (%)
Mother’s age	
18–24	1049 (9.1)
25–34	8180 (70.6)
≥35 years	2354 (20.3)
Nationality	
French	10,727 (92.6)
French by naturalisation	325 (2.8)
Foreigner	531 (4.6)
Educational level	
<9	1429 (12.3)
9–11	2081 (18.0)
≥12 years	8073 (69.7)
Mother’s employment status during pregnancy	
Employed or student	10,108 (87.3)
Housewife, on parental leave or retired	939 (8.1)
Unemployed	536 (4.6)
Number of children	
1	5296 (45.7)
2	4251 (36.7)
3 or more	2036 (17.6)
Familial financial status	
High and middle	6764 (58.4)
Low	4022 (34.7)
Very low	797 (6.9)
Psychiatric history	*n* (%)
Consultation with a mental health specialist before pregnancy (psychiatrist, psychologist, psychotherapist or another doctor)	
Yes	832 (7.2)
No	10,751 (92.8)
Depression during a previous pregnancy	
Yes	832 (7.2)
No	10,751 (92.8)

## Data Availability

The ELFE has an open-data policy after an 18-month exclusivity period following each release of new data. The data-access policy, study protocols, questionnaires and data catalogue can be found online: [https://www.ELFE-france.fr/en/].

## Supplementary Materials

The following supporting information can be downloaded at: https://www.mdpi.com/article/10.3390/jcm12093204/s1, Figure S1: Graphic representation of the eigenvalues of Perceived postnatal instrumental support; Figure S2: Graphic representation of the eigen values of Selfe-regulation skills; Table S1: Description of the sample (n = 11,583); Table S2: Characteristics of women with missing data (n = 6722); Table S3: Socio-demographic and economic variables correlation matrix; Table S4: Perceived antenatal emotional support from partner correlation matrix; Table S5: Eigenvalues of Perceived antenatal emotional support from partner correlation matrix; Table S6: Factor solution of Perceived antenatal emotional support from partner correlation matrix; Table S7: Perceived postnatal instrumental support correlation matrix; Table S8: Eigenvalues of Perceived postnatal instrumental support correlation matrix; Table S9: Factor solution of Perceived postnatal instrumental support after Varimax rotation; Table S10: Obstetrical complications correlation matrix; Table S11: Psychological factors correlation matrix; Table S12: Early maternal parenting behaviour correlation matrix; Table S13: Infant’s physical health matrix correlation; Table S14: Infants’ self-regulation skills correlation matrix; Table S15: Eigen values of Self-regulation skills correlation matrix; Table S16: Factor solution of Self-regulation skills.
