# Supplementary material for: Pathways between Risk/Protective Factors and Maternal Postnatal Depressive Symptoms: The ELFE Cohort
Source: J Clin Med. 2023 Apr 29;12(9):3204. doi: 10.3390/jcm12093204 (PMC10179307; doi:10.3390/jcm12093204)
Supplement: Supplementary file 1 [file jcm-12-03204-s001.zip › jcm-2275450-supplementary.pdf]

Table S1. Description of the sample (n=11,583)

**Maternal et environmental risk or protective factors**	
Perceived antenatal emotional support from partner	n (%)
Antenatal emotional support from spouse	
Very good support	6,155 (53.1)
Good support	4,543 (39.2)
Little support	776 (6.7)
No support	109 (0.9)
Quarrels with or without insults within the couple	
Never	2,184 (18.9)
Rarely	4,741 (40.9)
Sometimes without insults	2,606 (22.5)
Sometimes with insults	2,052 (17.7)
Perceived postnatal instrumental support	n (%)
For baby's care	
Changing diapers	
Mainly the mother	8,918 (77.0)
Mainly the father	65 (0.6)
Equal division of the labour	2,566 (22.2)
Another person	34 (0.3)
Not concerned	0 (0)
Feeding	
Mainly the mother	9,180 (79.3)
Mainly the father	37 (0.3)
Equal division of the labour	2,215 (19.1)
Another person	32 (0.3)
Not concerned	119 (1.0)
Wash	
Mainly the mother	7,553 (65.2)
Mainly the father	829 (7.2)
Equal division of the labour	3,187 (27.5)
Another person	14 (0.1)
Not concerned	0 (0)
Put it to sleep	
Mainly the mother	6,266 (54.1)
Mainly the father	466 (4.0)
Equal division of the labour	4,842 (41.8)
Another person	8 (0.1)
Not concerned	1 (0.0)
Taking it for a walk	
Mainly the mother	6,389 (55.2)
Mainly the father	94 (0.8)
Equal division of the labour	4,975 (43.0)
Another person	48 (0.4)
Not concerned	77 (0.7)

Getting up in the night because of cries	
Mainly the mother	8,283 (71.5)
Mainly the father	367 (3.2)
Equal division of the labour	2,748 (23.7)
Another person	3 (0.0)
Not concerned	182 (1.6)
Taking it to the doctor	
Mainly the mother	7,457 (64.4)
Mainly the father	60 (0.5)
Equal division of the labour	4,051 (35.0)
Another person	1 (0.0)
Not concerned	14 (0.1)
For household chores	
Washing dishes	
Mainly the mother	4,608 (39.8)
Mainly the father	1,437 (12.4)
Equal division of the labour	5,396 (46.6)
Another person	140 (1.2)
Not concerned	2 (0.0)
Doing shopping	
Mainly the mother	4,580 (39.5)
Mainly the father	2,312 (20.0)
Equal division of the labour	4,627 (39.9)
Another person	51 (0.4)
Not concerned	13 (0.1)
Preparing meals	
Mainly the mother	6,762 (58.4)
Mainly the father	1,607 (13.9)
Equal division of the labour	3,152 (27.2)
Another person	61 (0.5)
Not concerned	1 (0.0)
Doing laundry	
Mainly the mother	9,593 (82.8)
Mainly the father	377 (3.3)
Equal division of the labour	1,513 (13.1)
Another person	99 (0.9)
Not concerned	1 (0.0)
Doing the housework	
Mainly the mother	6,280 (54.2)
Mainly the father	646 (5.6)
Equal division of the labour	3,795 (32.8)
Another person	853 (7.4)
Not concerned	9 (0.1)
Repairs	
Mainly the mother	488 (4.2)
Mainly the father	8,967 (77.4)

Equal division of the labour	1,328 (11.5)
Another person	578 (5.0)
Not concerned	222 (1.9)

Maternal antenatal preventive measures	n (%)
Attending early prenatal interview, attending antenatal classes	
Attending the two	3,400 (29.4)
Attending one	4,675 (40.4)
Attending none	3,508 (30.3)

Obstetrical complications	n (%)
Complications during pregnancy	
None	4,964 (42.9)
Level 3	4,438 (38.3)
Level 4	2,148 (18.5)
Level 5 or 6	33 (0.3)
Complications at birth or neonatal complications	
None	1,452 (12.5)
Level 3	7,122 (61.5)
Level 4	1,653 (14.3)
Level 5 or 6	1,356 (11.7)

Psychological factors	n (%)
Reaction about the current pregnancy	
Happy that it happened now	8,923 (77.0)
Ambivalent towards this pregnancy	2,480 (21.4)
Unwanted pregnancy	180 (1.6)

Maternal et environmental risk or protective factors

Psychological factors	n (%)
Experience of pregnancy	
Pleasant	4,155 (35.9)
Pleasant with some difficulties	5,704 (49.2)
Difficult	1,724 (14.9)
Desire of pregnancy	
Yes	10,992 (94.9)
With hesitations	53 (0.5)
No	538 (4.6)
Prenatal psychological distress	
Yes	1,361 (11.8)
No	10,222 (88.3)

Infant characteristics and environmental factors specific to parenthood

Early maternal parenting behavior	n (%)
Maternal ability to understand infant’s cry	
Rarely	139 (1.2)
Sometimes	987 (8.5)
Often	10,457 (90.3)
Respect of infant’s feeding rhythm	

Wakes him to feed	277 (2.4)
Requests him at regular time but only if baby is awake	2,363 (20.4)
Feeds him on demand	8,943 (77.2)
Mother's reaction if baby eats little or don't finish his bottle (without illness context)	
Insists to feed him	1,103 (9.5)
Proposes him later	2,477 (21.4)
Don't insist or it never happens	8,003 (69.1)
Infants' physical health	
Infants' condition evaluated by the mother	
Healthy	10,054 (86.8)
Rather healthy	1,431 (12.4)
Rather poor health or poor health	98 (0.8)
Infant hospitalization since returning from maternity hospital stay	
Yes	672 (5.8)
No	10,911 (94.2)

**Infant risk or protective factors**	
Infants' self-regulation skills	n (%)
Self-appeasement	
Never or almost never	1,234 (10.7)
Often only	3,780 (32.6)
Only if parents remain with him	1,199 (10.4)
Only if parents take him in arms	5,370 (46.4)
Frequency of crying	
Rarely	7,629 (65.9)
Often	3,421 (29.5)
Very often	533 (4.6)
Nocturnal awakenings	
Never or almost never	3,948 (34.1)
Sometimes or often	2,041 (17.6)
All nights or almost	5,594 (48.3)

Table S2. Characteristics of women with missing data (n=6,722)

**Maternal and environmental vulnerability factors**	
Socio-demographic and economic variables	n (%)
Mother's age	
18-24	1333 (19.8)
25-34	3895 (57.9)
≥ 35 years	1299 (19.3)
Missing	195 (2.9)
Nationality	
French	5120 (76.2)
French by naturalisation	342 (5.1)
Foreigner	960 (14.3)
Missing	300 (4.5)
Educational level	
< 9	1,988 (29.6)
9-11	1,711 (25.5)
≥ 12 years	2,833 (42.1)
Missing	190 (2.8)
Mother's employment status during pregnancy	
Employed or student	4,096 (60.9)
Housewife, on parental leave or retired	492 (7.3)
Unemployed	1,138 (16.9)
Missing	996 (14.8)
Number of children	
1	3,022 (45.0)
2	1,993 (29.6)
3 or more	1,426 (21.2)
Missing	281 (4.2)
Familial financial status	
High and middle	2,104 (31.3)
Low	1,773 (26.4)
Very low	646 (9.6)
Missing	2,199 (32.7)
Psychiatric history	n (%)
Consultation with a mental health specialist before pregnancy (psychiatrist, psychologist, psychotherapist or another doctor)	
Yes	603 (9.0)
No	1,820 (27.1)
Missing	4,299 (64.0)
Depression during a previous pregnancy	
Yes	447 (6.6)
No	5,907 (87.9)
Missing	368 (5.5)

Table S3. Socio-demographic and economic variables correlation matrix

	Mother's age	Nationality	Educational level	Mother's employment status during pregnancy	Familial financial status	Number of children
**Mother's age**	1.00	0.04	-0.22	0.09	-0.05	0.31
**Nationality**		1.00	0.02	0.12	0.05	0.02
**Educational level**			1.00	0.25	0.25	0.13
**Mother's employment status during pregnancy**				1.00	0.14	0.13
**Familial financial status**					1.00	0.11
**Number of children**						1.00

KMO = 0.52

Table S4. Perceived antenatal emotional support from partner correlation matrix

	Antenatal emotional support from spouse	Quarrels with or without insults
**Antenatal emotional support from spouse**	1.00	0.31
**Quarrels with or without insults**		1.00

Table S5. Eigenvalues of Perceived antenatal emotional support from partner correlation matrix

	Eigenvalues	Difference	Variance explained	Cumulative
1	1.31	0.62	0.65	0.65
2	0.69		0.35	1.00

Table S6. Factor solution of Perceived antenatal emotional support from partner correlation matrix

Représentation du facteur	
	**Factor 1**
**Antenatal emotional support from spouse**	0.81
**Quarrels with or without insults**	0.81

Table S7. Perceived postnatal instrumental support correlation matrix

	Changing diapers	Feeding	Put to bed	Wash	Taking for a walk	Getting up in the night	Taking it to the doctor	Washing dishes	Doing shopping	Preparing meals	Doing laundry	Doing the housework	Repairs
**Changing diapers**	1.00	0.48	0.29	0.27	0.28	0.26	0.20	0.12	0.06	0.09	0.11	0.10	0.01
**Feeding**		1.00	0.27	0.11	0.27	0.28	0.18	0.05	-0.001	0.05	0.05	0.02	-0.002
**Put to bed**			1.00	0.29	0.19	0.30	0.15	0.10	0.04	0.06	0.07	0.07	0.01
**Wash**				1.00	0.12	0.12	0.16	0.09	0.07	0.09	0.08	0.12	0.02
**Taking for a walk**					1.00	0.16	0.25	0.06	0.12	0.10	0.08	0.04	0.01
**Getting up in the night**						1.00	0.14	0.09	0.01	0.05	0.07	0.06	-0.002
**Taking it to the doctor**							1.00	0.04	0.19	0.08	0.08	0.03	0.02
**Washing dishes**								1.00	0.13	0.21	0.23	0.27	0.02
**Doing shopping**									1.00	0.25	0.14	0.10	0.03
**Preparing meals**										1.00	0.10	0.11	0.02
**Doing laundry**											1.00	0.20	0.04
**Doing the housework**												1.00	0.05
**Repairs**													1.00

KMO = 0.74

Table S8. Eigenvalues of Perceived postnatal instrumental support correlation matrix

	Eigenvalues	Difference	Variance Explained	Cumulative
**1**	2.60	1.05	0.19	0.19
**2**	1.55	0.41	0.11	0.30
**3**	1.14	0.11	0.08	0.38
**4**	1.03	0.05	0.07	0.45
**5**	0.98	0.03	0.07	0.52
**6**	0.95	0.05	0.07	0.59
**7**	0.90	0.07	0.06	0.65
**8**	0.83	0.05	0.06	0.71
**9**	0.78	0.05	0.06	0.77
**10**	0.73	0.02	0.05	0.82
**11**	0.71	0.03	0.05	0.87
**12**	0.68	0.05	0.05	0.92
**13**	0.63	0.15	0.05	0.97
**14**	0.48		0.03	1.00

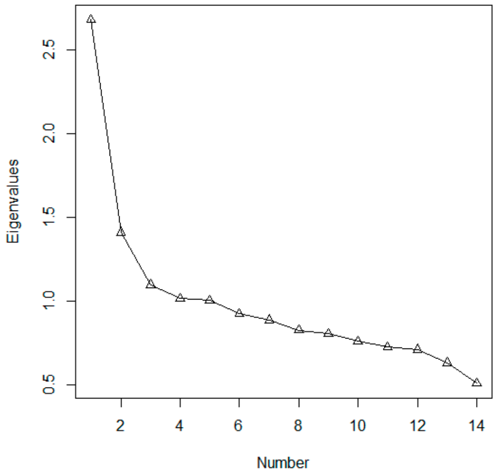

Figure S1. Graphic representation of the eigenvalues of Perceived postnatal instrumental support

Table S9. Factor solution of Perceived postnatal instrumental support after Varimax rotation

	Factor 1	Factor 2
**Changing diapers**	0.73	0.05
**Feeding**	0.70	-0.12
**Put to bed**	0.61	0.03
**Wash**	0.44	0.18
**Taking for a walk**	0.54	0.08
**Getting up in the night**	0.57	-0.02
**Taking it to the doctor**	0.43	0.15
**Wash**	0.11	0.63
**Doing shopping**	0.06	0.54
**Preparing meals**	0.10	0.54
**Doing laundry**	0.10	0.54
**Doing the housework**	0.06	0.58
**Repairs**	-0.01	0.15

Table S10. Obstetrical complications correlation matrix

	Complications during Pregnancy	Complications at Birth Or Neonatal Complications
**Complications during pregnancy**	1.00	0.11
**Complications at birth or neonatal complications**		1.00

Table S11. Psychological factors correlation matrix

	Desire of Pregnancy	Reaction about the Current Pregnancy	Experience of Pregnancy
**Desire of pregnancy**	1.00	0.23	0.07
**Reaction about the current pregnancy**		1.00	0.09
**Experience of pregnancy**			1.00

KMO =0.53

Table S12. Early maternal parenting behaviour correlation matrix

	Sing songs	Talk o the child	Maternal ability to understand infant's cries	Respect of infant's feeding rhythm	Mother's reaction if baby eats little or don't finish his bottle
**Sing songs**	1,00	0,08	0,02	0,03	0,01
**Talk o the child**		1,00	0,03	-0,00	0,02
**Maternal ability to understand infant's cries**			1,00	0,01	0,03
**Respect of infant's feeding rhythm**				1,00	0,08
**Mother's reaction if baby eats little or don't finish his bottle**					1,00

KMO =0.52

Table S13. Infant's physical health matrix correlation

	Infant's condition evaluated by the mother	Infant's hospitalisation since returning from maternity hospital
**Infant's condition evaluated by the mother**	1.00	0.26
**Infant's hospitalisation since returning from maternity hospital**		1.00

Table S14. Infants' self-regulation skills correlation matrix

	Self-Appeasement	Frequency of Crying	Nocturnal Awakenings
**Self-appeasement**	1.00	0.29	0.18
**Frequency of crying**		1.00	0.18
**Nocturnal awakenings**			1.00

KMO = 0.57

Table S15. Eigen values of Self-regulation skills correlation matrix

	Eigen values	Difference	Variance Explicated	Cumulative
**1**	1.50	0.36	0.25	0.25
**2**	1.14	0.12	0.19	0.44
**3**	1.02	0.16	0.16	0.61
**4**	0.86	0.05	0.14	0.75
**5**	0.80	0.11	0.13	0.89
**6**	0.69		0.11	1.00

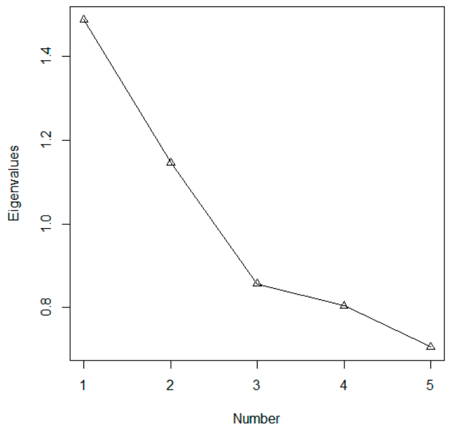

Figure S2. Graphic representation of the eigen values of Self-regulation skills

Table S16. Factor solution of Self-regulation skills

	Facteur 1
Self-appaisement	0,68
Frequency of crying	0,66
Nocturnal awakenings	0,60
